# Real-time Detection and Monitoring of Loop Mediated Amplification (LAMP) Reaction Using Self-quenching and De-quenching Fluorogenic Probes

**DOI:** 10.1038/s41598-018-23930-1

**Published:** 2018-04-03

**Authors:** Vijay J. Gadkar, David M. Goldfarb, Soren Gantt, Peter A. G. Tilley

**Affiliations:** 1 Department of Pathology & Laboratory Medicine, Division of Microbiology, Virology & Infection Control, Children’s & Women’s Health Center of British Columbia, 4500 Oak St, Vancouver, V6H 3N1 Canada; 2 Department of Pediatrics, Division of Infectious Diseases, University of British Columbia and Children’s & Women’s Health Center of British Columbia, 4500 Oak St, Vancouver, V6H 3N1 Canada

## Abstract

Loop-mediated isothermal amplification (LAMP) is an isothermal nucleic acid amplification (iNAAT) technique known for its simplicity, sensitivity and speed. Its low-cost feature has resulted in its wide scale application, especially in low resource settings. The major disadvantage of LAMP is its heavy reliance on indirect detection methods like turbidity and non-specific dyes, which often leads to the detection of false positive results. In the present work, we have developed a direct detection approach, whereby a labelled loop probe quenched in its unbound state, fluoresces only when bound to its target (amplicon). Henceforth, referred to as Fluorescence of Loop Primer Upon Self Dequenching-LAMP (FLOS-LAMP), it allows for the sequence-specific detection of LAMP amplicons. The FLOS-LAMP concept was validated for rapid detection of the human pathogen, Varicella-zoster virus, from clinical samples. The FLOS-LAMP had a limit of detection of 500 copies of the target with a clinical sensitivity and specificity of 96.8% and 100%, respectively. The high level of specificity is a major advance and solves one of the main shortcomings of the LAMP technology, i.e. false positives. Self-quenching/de-quenching probes were further used with other LAMP primer sets and different fluorophores, thereby demonstrating its versatility and adaptability.

## Introduction

Challenges in exploiting polymerase chain reaction (PCR) for point-of-care (POC) diagnostics have resulted in the development of nucleic acid amplification tests based on isothermal principles that are collectively referred to as iNAAT’s^1–3^. Examples of such technologies include: strand-displacement amplification (SDA)^4^, helicase-dependent amplification (HDA)^5^, recombinase polymerase amplification (RPA)^6^ and loop-mediated amplification (LAMP)^7^. One advantage of iNAAT’s over PCR is that they amplify their targets at a constant temperature, thereby obviating the time consuming thermocycling steps required for any two- or three-step PCR. As no thermocycling steps are performed such, iNAAT reactions are extremely fast (5–20 min), resulting in shorter “sample-to-answer” times than conventional PCR^8^. With no thermocycling requirements, amplification reactions can be conducted using simple instruments – e.g., a conventional water bath or heat block – drastically reducing equipment costs. This particular feature has made isothermal amplification systems an attractive platform for POC use and in resource-limited markets^9,10^.

Amongst the iNAAT’s, the LAMP technology *per se*, has found wide scale application in both laboratory and POC diagnostics^11^. This *in vitro* amplification technique^7^, carried out anywhere between 60–65 °C, results in the amplification of a few target molecules to 10^9^–10^10^ copies. Due to the high rate of product formation (>10 µg, >50X PCR yield) within a short period of time (10–15 minutes), LAMP has detection sensitivity comparable to or in many cases, exceeding that of real-time quantitative PCR (qPCR)-the most common “gold standard” technique in most molecular diagnostic assays. The LAMP system principally employs four core primers, namely FIP (forward inner primer), BIP (backward inner primer), F3 (forward primer) and B3 (backward primer) to recognize six different regions of the target sequences. Two optional primers, LF (loop forward) and LB (loop backward), can be added to the amplification reaction to enhance the reaction speed. This modification, also known as “accelerated LAMP”^12^, was a later modification of the classical, four-primer LAMP^7^. Though the exact mechanism is unclear, the LF/LB primers presumably accelerate the four primer LAMP reaction by creating additional binding sites for the auto-cycling FIP/BIP primers^12^. The auto-cycling leads to the formation of “cauliflower-like” DNA structures, which essentially are DNA concatamers with loops interspersed between alternating inverted repeats^7^. Formation of these multimeric products of the target region represents a successful amplification of the target DNA^7^.

Typically, the measurement of LAMP products relies on end-point analysis and requires post-amplification processing, leading to possible cross-contamination or detection of non-specific LAMP amplicons. Some of these methods include: resolving amplified products on agarose gel electrophoresis^7^, turbidity analysis of positive reactions due to the accumulation of magnesium pyrophosphate (Mg_2_P_2_O_7_)^13^, detection of dsDNA under UV-light in presence of an intercalating dyes like SYBR Green I or EvaGreen^14^ and addition of metal ion indicators like, calcein/Mn^2+^ and hydroxynapthol blue dye (HNB)^15^. Amongst these, the use of intercalating fluorescent dyes has been favoured for clinical diagnostics as they are more sensitive and relatively tolerant towards opaque substances like proteins, which are known to affect turbidimetric signal^16^. A major disadvantage, however, of using non-specific detection methods is the increased likelihood of detecting false positives. This is despite the fact that LAMP relies on 4–6 different primers to independently recognize 6–8 independent regions on the target sequence suggesting, at least in theory, a higher degree of specificity than a two-primer PCR. Though the mechanism of non-specific amplification remains unclear, it is assumed that *cis* and *trans* priming amongst the six primers, could be responsible for this phenomenon^17^. Thus, indirect detection of amplification products remains one of major shortcomings of LAMP technology.

Fluorophores labelled nucleic acids, that specifically hybridize, in a sequence dependent manner, to a transiently generated single-stranded DNA structure, have proven to be an ideal solution to any non-specific, dye-based detection system. Examples of such include the, hydrolysis-based TaqMan™ probes specifically developed for qPCR^18^ and molecular beacons^19^ among host of others. Due to the atypical amplification chemistry of iNAAT’s and LAMP *per se*, seamless application of any of these probe technologies, specifically developed for qPCR have proven to be technically challenging. Attempts however have been made to develop a probe-based detection system for LAMP include: loss-of-signal guanine quenching^20^, gain-of-signal fluorescence using labeled primers^21^, detection of amplification by release of quenching (DARQ)^22^, assimilating probe^23^, one-step-toe-hold (OSD) reporter^24^ and more recently, molecular beacons^25^.

Here we demonstrate the use of self-quenching fluorogenic probes as an alternative approach to detect and monitor LAMP reactions in real-time. These probes, originally developed as part of luminescence upon extension technology^26,27^, were tested for their suitability to detect LAMP reaction products in real-time. This new real-time LAMP technology, henceforth, referred to as Fluorescence of Loop Primer Upon Self Dequenching-LAMP (FLOS-LAMP), was optimized to detect a clinically important human pathogen, Varicella-zoster virus (VZV).

The test target virus VZV selected for optimization is the causative agent of chicken pox and herpes zoster, often results in central nervous system manifestations and pneumonia in both immunecompetent and immunocompromised individuals. These alphaherpesviruses infect and establish lifelong latency, subsequently reactivating from human sensory neuronal ganglia. Its rapid and early detection is very important in symptomatic patients from both treatment and infection control view point. Due to its unique signal development mechanism i.e., fluorescence only in the presence of its target, the presented LAMP methodology greatly improves our ability to specifically detect VZV in actual clinical samples.

## Results

### Design of FLOS-LAMP probes

Among each of the three functional categories currently defined for a six-primer LAMP, namely outer, inner and loop, we could identify at least one candidate primer for labelling (Table 1). Specifically, the VZV62F3 primer was identified in the outer primer category while, VZV62BIP, VZV62FIP and VZV62LPB were identified in the inner and loop categories, respectively (Table 1). From the inner primer category, only the VZV62FIP primer was submitted for labelling.

**Table 1:** The VZV62 LAMP Primer set. T denotes the site for FAM attachment.

Target	Primer			Sequence
	Category	Sub-category	Name	
Varicella zoster virus (*ORF62*)	**Outer**	Outer Forward	VZV62F3	TCAGAAGCCTCACATCC**T**CC
		Outer Reverse	VZV62B3	CGTACTGTACCCCCGAAAC
	**Inner**	Inner Backward	VZ62BIP	GGCGCCGGGATCAAAGCTTATTTTGGTCGACGACCCATTGT**T**TC
		Inner Forward	VZ62FIP	GACGGTTTGGTCCACCCAGCTCTGGGATCTGCCGCA**T**C
	**Loop**	Loop Forward	VZ62LPF	AGTGGAGGCGCTGCGACGGA
		Loop Reverse	VZ62LPB	CGCAGGGCGCCAGGCCG**T**GG

### Isothermal Amplification of pVZV-ORF62 test template

**Figure 1:**
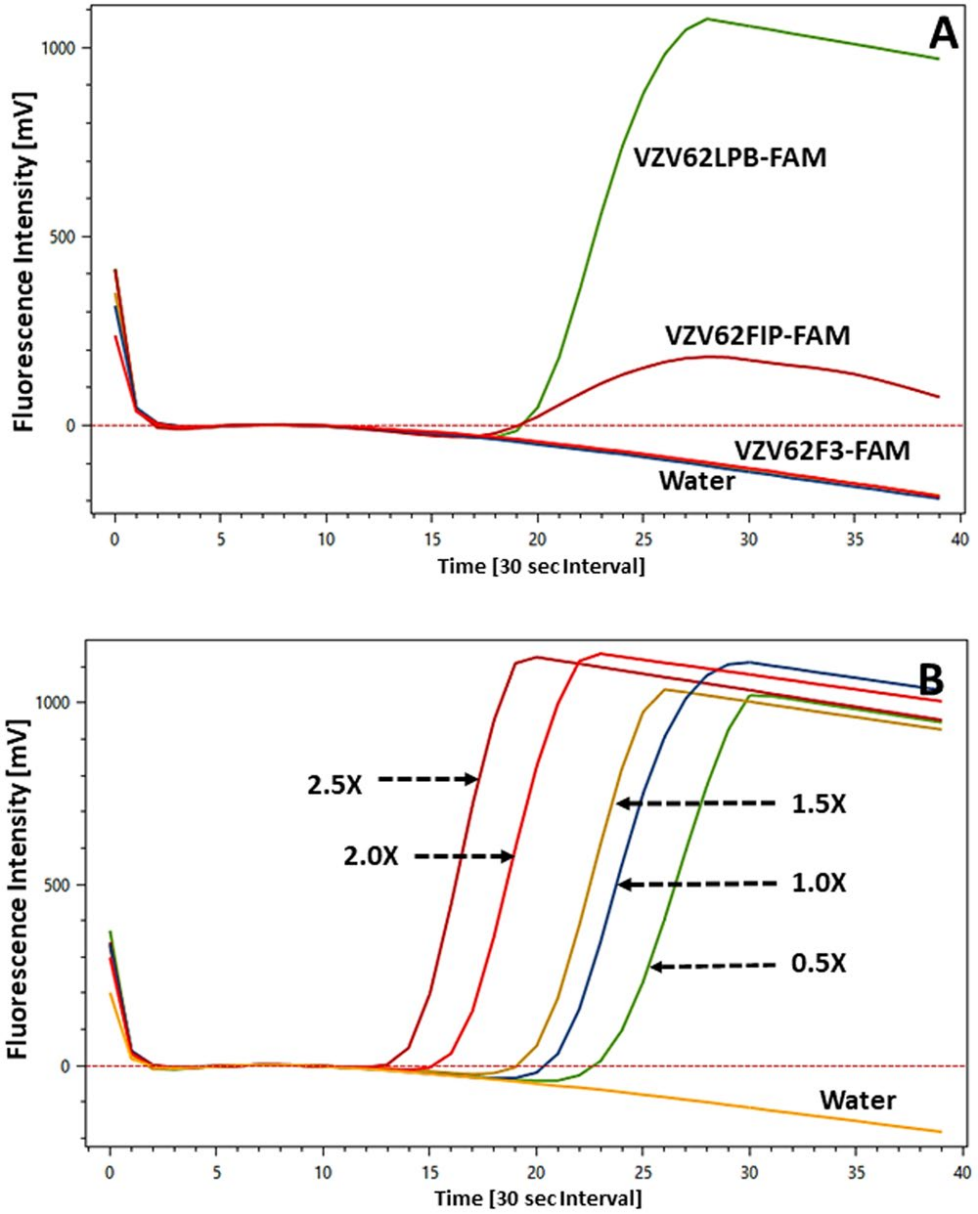
Fluorescence signal from VZV62 dT-FAM probes. (**A**) Signal profile obtained after converting the Outer (VZV62F3), Inner (VZV62FIP) and Loop (VZV62LPB) primers into dT-FAM fluorescent probes. Input template concentration pVZV-ORF62 plasmid: 1 × 10^6^ copies/µL. (**B**) Variation of fluorescence signal intensity due to varying levels of the Base Mix ranging from 0.5X, 1.0X, 1.5X, 2.0X and 2.5X. Input template concentration pVZV-ORF62 plasmid: 1 × 10^6^ copies/µL. Water was used as a negative control. Each Time[30 sec interval] unit (X-axis) represents 30 seconds on the ESEQuant instrument.

When the FAM-labelled VZV62F3 primer was used to amplify the test template, pVZV-ORF62 plasmid (1 × 10^6^ copies/µL), no signal could be detected (Fig. 1A). Changes in the concentration of either the probe (0.1 µM to 0.3 µM) or unlabelled primers (VZV62F3/VZV62B3/VZV62FIP/VZV62BIP/VZV62LPF) did not alter this negative result. However, when VZV62FIP-FAM probe (0.2 µM) was used with the same plasmid template (pVZV-ORF62: 1 × 10^6^ copies/µL), a positive amplification curve (Tp = 9.5 min) was obtained (Fig. 1A). The water control of this probe/primer combination did not result in any fluorescence above baseline (signal <0 mV), indicating that the observed positive signal was due to the incorporation of the VZV62FIP-FAM probe into the reaction product. For the concentration of the VZV62FIP-FAM probe (0.2 µM) used, the maximum intensity of the amplification signal varied between 200 to 250 mV (Fig. 1A). Any increase in concentration of the VZV62FIP-FAM probe to 0.3 µM and manipulation of other unlabelled primers, did not alter either the Tp (varying 9–9.5 min) or the amplitude of the fluorescence signal (200 to 250 mV). Interestingly, when the labelled VZV62LPB-FAM (0.2 µM) probe was used instead, the signal amplitude became four times higher (1000–1100 mV) than the VZV62FIP-FAM probe (200 to 250 mV), under similar template input conditions (pVZV-ORF62: 1 × 10^6^ copies/µL). Due to the high signal amplitude obtained using VZV62LPB-FAM, further optimization was pursued using this probe.

### Optimization of VZV62LPB-FAM signal

#### Base Mix

The concentration of unlabelled primers used with the VZV62LPB-FAM probe (0.2 µM) was determined using the “Base Mix” (Table 2). This primer concentration range, recommended by the manufacturer (Optigene) for the dye based ISO-004 mastermix, served as the starting point for manipulating individual unlabelled primers (VZV62F3, VZV62B3, VZV62FIP, VZV62BIP, VZV62LPF). Increasing the Base Mix from 1X to 1.5X, 2X and 2.5X levels, resulted in a decrease of Tp from 9.5 minutes (1X Base Mix) to 6.5 minutes (2.5X Base Mix; Fig. 1B). Limited testing with higher reaction volumes, to accommodate the increased volume (>2.5X) of the Base Mix, resulted in no significant change in Tp rate and a distinct reduction in signal amplitude (<400 mV) was regularly observed (data not shown). The concentrations of individual primers obtained in 2.5X of the Base Mix (Table 2), were used for further optimization.

**Table 2:** Varying concentrations of the Base Mix used in VZV62 FLOS-LAMP assay.

Primer Name	0.5X	Base Mix	1.5X	2.0X	2.5X
VZV62F3	0.1 µM	**0.2 µM**	0.3 µM	0.4 µM	0.5 µM
VZV62B3	0.1 µM	**0.2 µM**	0.3 µM	0.4 µM	0.5 µM
VZ62BIP	0.4 µM	**0.8 µM**	1.2 µM	1.6 µM	2.0 µM
VZ62FIP	0.4 µM	**0.8 µM**	1.2 µM	1.6 µM	2.0 µM
VZ62LPF	0.2 µM	**0.4 µM**	0.6 µM	0.8 µM	1.0 µM

#### Manipulation of F3, B3 levels

**Figure 2:**
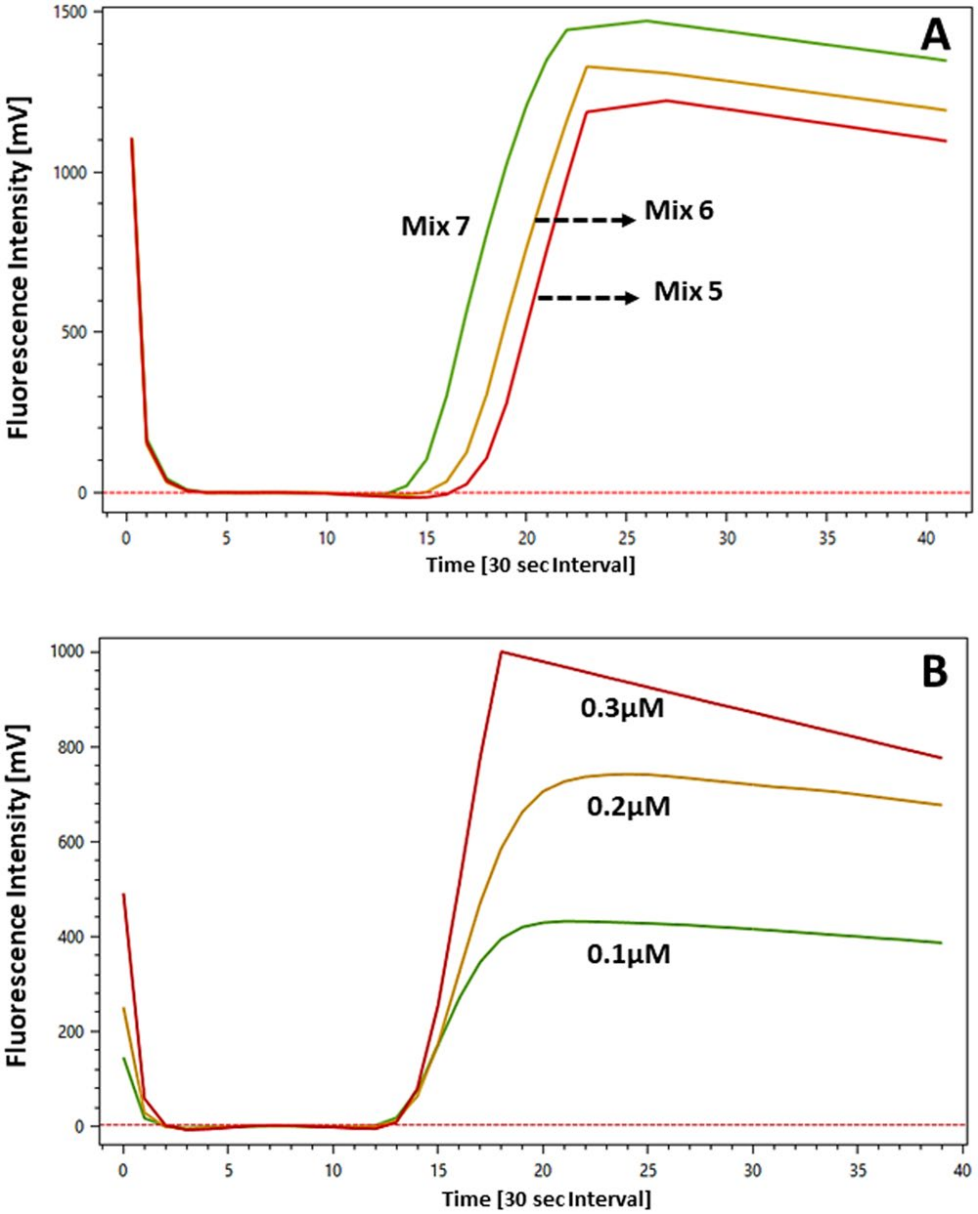
Optimization of signal intensity using the VZV62LPB-FAM probe. (**A**) Effect of increasing levels of F3/B3 primer pair from 0.5 µM (Mix 5), 1.0 µM (Mix 6) and 2.0 µM (Mix 7_OPT_). (**B**) Variation in signal intensity with increasing levels of the VZV62LPB-FAM probe from 0.1 µM, 0.2 µM and 0.3 µM. Input template concentration of pVZV-ORF62 plasmid: 1 × 10^6^ copies/µL. Each Time[30 sec interval] unit (X-axis) represents 30 seconds on the ESEQuant instrument.

As any increase in the Base Mix concentration >2.5X was found to adversely affect the Tp therefore, the concentration of the inner primers (VZV62FIP/VZV62BIP) was maintained at 2.0 µM (Mix 5; Table 3). Increasing the individual concentrations of the outer primers (VZV62F3/VZV62B3), resulted in a slight decrease in Tp (Fig. 2A), with highest amplitude noted at 2.0 µM concentration level (Mix 7; Table 3). Mix 7 therefore contained an equimolar concentration (2.0 µM) of outer and inner primers. Attempts to change the concentration of the loop primer (VZV62LPF) above 1.0 µM levels (Mix 5; Table 3) had an overall negative effect on Tp. Therefore, the concentration of this primer was maintained at 1.0 µM. The high amplitude, equimolar Mix 7 henceforth referred to as Mix 7_OPT_, was considered to be the optimal primer mixture and used in further studies. Limited testing with higher volumes of the Mix 7_OPT_ mix, specifically, 1.5X and 2X did not result in any further decrease in Tp, compared to the 1X Mix 7_OPT_ (See Supplementary Fig. S1). In other words, further increase in the concentration of Mix 7_OPT_ does not speedup the reaction but one does see a decrease in signal amplitude.

**Table 3:** Variation of F3/B3 primer concentration in the unlabelled VZV62 primer formulation.

Primer Name	Mix 5 (2.5X Base Mix)	Mix 6	Mix 7_OPT_
VZV62F3	0.5 µM	1.0 µM	2.0 µM
VZV62B3	0.5 µM	1.0 µM	2.0 µM
VZ62BIP	2.0 µM	2.0 µM	2.0 µM
VZ62FIP	2.0 µM	2.0 µM	2.0 µM
VZ62LPF	1.0 µM	1.0 µM	1.0 µM

#### Probe concentration

The effect of different concentrations of the VZV62LPB-FAM probe on the final signal development was tested. Increasing the VZV62LPB-FAM probe from 0.1 µM to 0.3 µM had a direct effect on the signal development (Fig. 2B). The signal amplitude progressively increased with increasing concentration of the probe however no change in Tp was observed. Concentrations >0.3 µM exceeded the detection range of the ESEQuant TS2.6 instrument.

### Limit of detection

A ten-fold serial dilution of the pVZV-ORF62 plasmid template showed that the VZV62 FLOS-LAMP was sensitive to 1.0 × 10^2^ copies/µL. This amounted to 500 copies per 20 µL reaction volume (Table 4). No signal was detectable below this concentration of target. In contrast, the dye based detection system could only detect the pVZV-ORF62 plasmid template down to 1.0 × 10^3^ copies/µL level (5,000 copies/20 µL reaction volume; Table 4). The average Tp values of the VZV62 FLOS- and dye-based detection LAMP were 9.5 min and 13.0 min, respectively.

**Table 4:** Comparison of limit of detection of the VZV62 FLOS-LAMP with intercalating dye based LAMP assay. nd = not detected.

pVZV-ORF62 (copies/µL)	Copies/Reaction (20 µL)	Time to positivity (Tp)	
		Flos-Lamp Probe	Intercalating Dye
1.0 × 10^5^	5,00,000	7.5 min	12.5 min
1.0 × 10^4^	5,00,00	8.5 min	14.0 min
1.0 × 10^3^	5,000	10.0 min	15.5 min
1.0 × 10^2^	500	14.5 min	nd
1.0 × 10^1^	50	nd	nd

### Performance of FLOS-LAMP on clinical samples

**Figure 3:**
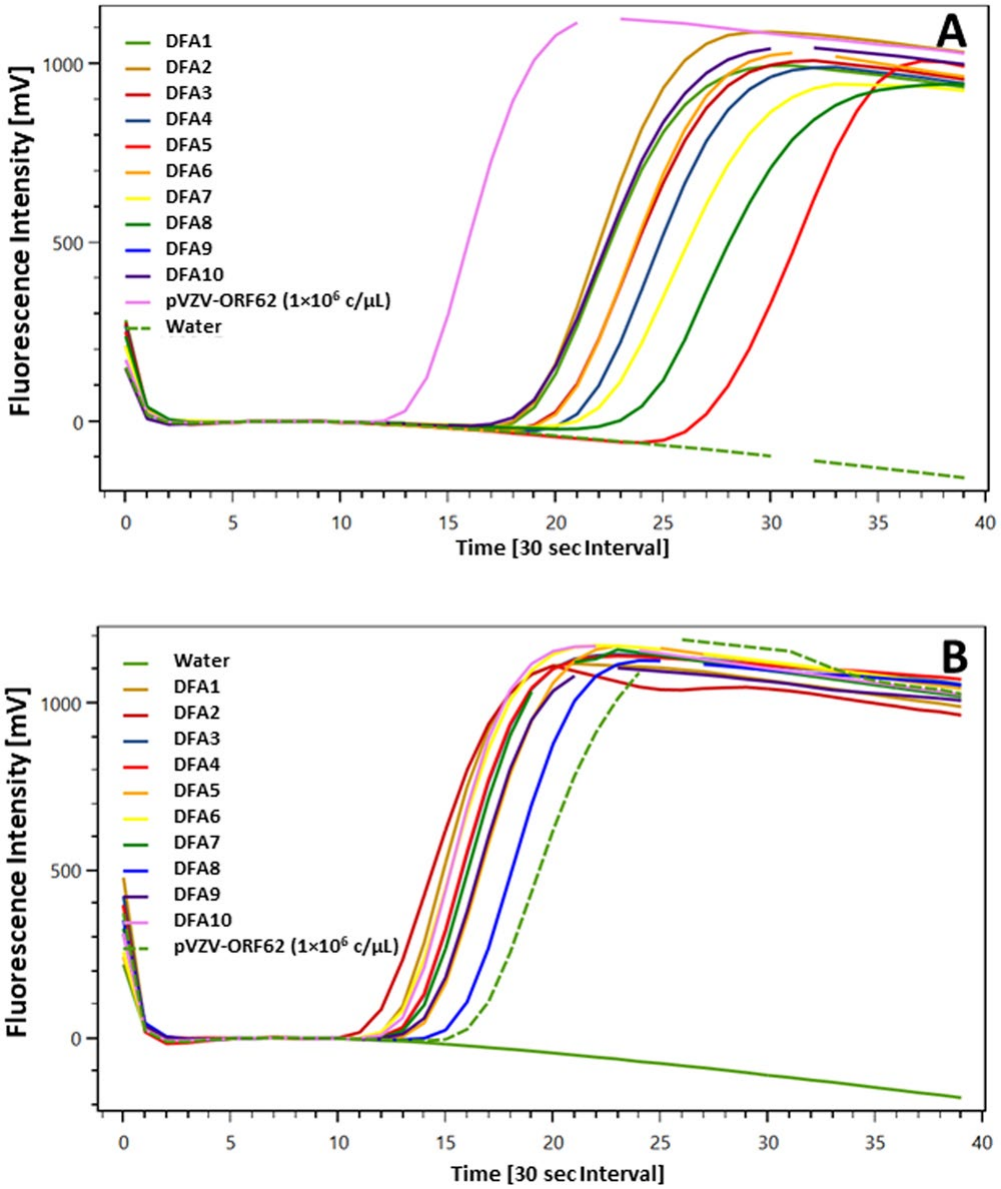
Detection of VZV from DFA positive clinical samples using VZV62 FLOS-LAMP assay. VZV DFA-positive clinical samples detected by the VZV62 FLOS-LAMP assay when (**A**) clinical samples were added directly into the reaction mixture and (**B**) after rapid DNA extraction using InstaGene™ matrix. Each Time[30 sec interval] unit (X-axis) represents 30 seconds on the ESEQuant instrument.

In all cases, addition of 5 µL of each DFA-positive VZV sample (n = 10) directly into the VZV62 FLOS-LAMP reaction mixture, resulted in a positive detection signal (Fig. 3A). When the same set of 10 samples were submitted for DNA extraction, which consisted of a simple heat lysis in presence of an extraction resin (InstaGene™), a more robust detection signal was obtained (Fig. 3B). This was reflected in a lower Tp value (avg Tp = 6.5 min) when compared to a direct specimen addition (no pre-extraction protocol (avg Tp = 11.1 min). Of the 22 VZV clinical samples that tested positive by qPCR (see Supplementary Table S4), all tested positive using the VZV62 FLOS-LAMP assay (see Supplementary Fig. S2) except for one sample, VZ12 (Cq = 39.8). Testing of non-target clinical (n = 34) samples did not result in any positive signals (see Supplementary Fig. S3). In addition, all clinical samples (n = 18) that previously tested negative for VZV by qPCR were also negative by VZV62 FLOS-LAMP (data not shown). Taking into consideration all the VZV-positive (n = 32) and -negative (n = 52) samples tested, the overall sensitivity and specificity of the VZV62 FLOS-LAMP assay was 96.8% (95% CI: 83.78% to 99.92%) and 100% (95% CI: 93.15% to 100.0%), respectively.

### FLOS-LAMP using HSV1-US4, pUC19 & CMV-UL54 primer sets

Modification of loop primers in other LAMP primer sets namely, CMV-UL54, HSV1-UL4 and pUC19 also gave positive results. Using concentration of respective unlabelled primers similar to Mix 7_OPT_, the CMV-UL54, pUC19, and HSV1-US4 sets generated positive fluorescence signal when tested against the respective templates while, no signal was detected from water controls (see Supplementary Fig. S4). This indicated that these self-quenched FLOS probes bind and fluoresce only in the presence of their respective targets. While the optimal concentration for FAM-based probes (UL54LB; HS1LPF) ranged from 0.15 to 0.3 µM, a much lower concentration (0.02–0.05 µM) was required for both JOE and ROX probes (pUC19A4LB).

## Discussion

LAMP is a DNA amplification technique that is attractive for molecular diagnostic assays, especially for POC use^11^. The advantages of this technique are that it is: (1) isothermal, and therefore requires no expensive thermocycling equipment, (2) rapid (Tp of 10–20 min) because no temperature ramping is required and, most importantly, (3) extremely sensitive, comparing favorably or, in some cases, better than qPCR^28^. However, like other isothermal amplification systems, LAMP is plagued by false positive signals being detected in negative control reactions^29^, which has hampered its use in clinical diagnostics. The generation of false positive is observed despite the fact that LAMP makes use of 4–6 primers to recognize 6–8 independent target sequences which in theory, should be providing higher specificity than standard two-primer PCR. The development FLOS-LAMP is an attempt to develop a more direct approach which can robustly detect the LAMP amplicon from its non-template background signal.

**Figure 4:**
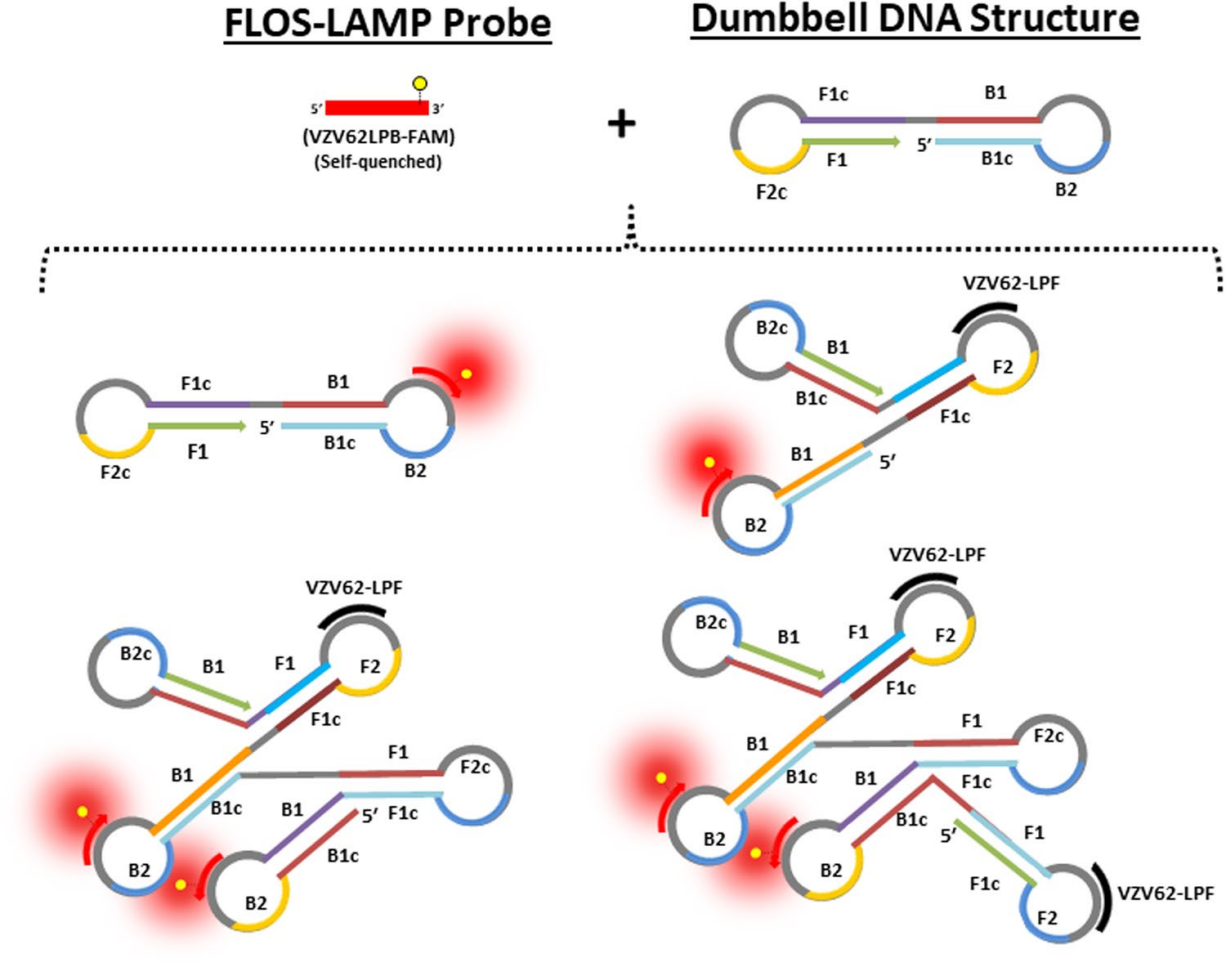
Schematic depiction of the binding of VZV62LPB-FAM probe to its target. The FAM fluorophore attached to the VZV62LPB-FAM probe is self-quenched in unbound state. Post binding to the dumbbell shaped DNA target, the FAM fluorophore is de-quenched resulting in fluorescence development. For simplicity, only one dumbbell which would bind to the LPB loop primer (VZV62LPB) is shown in this depiction.

A fluorescent probe-based detection technology, whereby a labelled oligonucleotide selectively binds to its target, resulting in target-specific fluorescence, has proven to be technically challenging to implement in LAMP. This is due to various factors which include (a.) the strand displacing polymerase enzyme lacking the 5′ to 3′ exonuclease activity and (b.) no temperature cycling, for discrete probe binding/dissociation stages. Probe based strategies proposed for LAMP to date^20–25^, have had one strategy in common- a FRET based approach. In this approach, a dedicated quencher moiety, present in close proximity to the fluorophore, has to be physically displaced for the flurophore to de-quench. In contrast, we have relied on a much simpler, non-FRET based approach whereby, the fluorophore’s quenches/de-quenches in absence of a dedicated quencher (Fig. 4). As no dedicated quencher is used in FLOS-LAMP, our strategy is not only cost-effective but also extremely simple to implement. This is because, some of the FRET-based strategies like DARQ^22^ and OSD^24^, require precise, pre-analytical steps like *in vitro* annealing of the fluorophore/quencher strands in equimolar amounts, to prepare the working probe^30^. Such pre-analytical step can be technically challenging for a laboratory with limited resources and/or technical expertise. No such complex pre-analytical steps are required while preparing FLOS-LAMP probes. This in our opinion is one of the major advantages of our FLOS-LAMP probe technology.

In the absence of any prior knowledge on the implementation of self-quenching probes in LAMP, we first experimented with attaching the fluorescent (FAM) label to at least one primer in each of the three functional categories (outer, inner and loop), currently defined for a six-primer LAMP assay (Table 1). While no signal was detected when the outer primer (VZV62F3) was labelled, a discernible positive signal was detected when labelled inner (VZV62FIP) or loop (VZV62LPF) primers were separately tested. Lack of signal using the F3 primer could be best explained by the functional role outer primers play during the amplification process. Specifically, the outer primers function as “displacing primers”, whereby they trigger the physical displacement of the FIP/BIP strands leading to its fold back which in subsequent steps, leads to the formation of the signature, dumbbell shaped, double-stranded DNA structure. In other words, outer primers are not actively incorporated into the final amplicon and therefore play only a minor role in the LAMP amplification cascade. This is consistent with the usage of outer primers at the lowest concentration in a classical dye-based LAMP assay. The positive signal generated by inner (VZV62FIP) and loop (VZV62LPF) primers (Fig. 1), is expected as primers in these categories are actively incorporated into the end product. However, markedly different signal amplitude of 200–250 mV (VZV62FIP) vs and 1000–1100 mV (VZV62LPF), between the probes of these two functional categories was observed (Fig. 1). While it is difficult to explain the consistent low amplitude of the VZV62FIP probe signal, one can only speculate upon the reason behind this observed phenomenon. Considering the fact that the inner primers (FIP/BIP) participate in the amplification cascade by repeatedly forming self-folding structures^7^, the attachment of a chemical moiety (FAM) to the nucleotide backbone might be imposing a steric hindrance, leading to its inefficient incorporation. On the other hand, the loop primer (VZV62LPF), during its binding is not required to fold back, and is thereby less susceptible to any adverse effect(s) of internal fluorophore attachment. Although it’s beyond the scope of the present work to elucidate the exact mechanism(s) through which the FAM flurophore, attached internally to the loop primer, is quenched/de-quenched, it’s most probably due to the interactive effect of certain nucleobases^26,27^. Of the four, guanosine has been shown to be an efficient electron donor whose presence (at 5′ end), near a flurophore can decrease its fluorescence upon hybridization^31^. Nazarenko *et al*.^26^ showed that if the flurophore is conjugated internally, the fluorescent property of primary and secondary deoxyoligonucleotide structure can change (10-fold) upon hybridization. Although the mechanism by which an internally conjugated label gets de-quenched remains unclear, it’s believed that guanosine which, has the highest electron donating ability of all bases might be playing a critical role in this process. This in turn is also dependent on the whether the nucleobase is involved in hydrogen bonding and whether it’s located at the end of the stranded structure or internally^26,27^.

Retrospective testing of VZV-positive clinical samples using the optimized VZV62 FLOS-LAMP assay showed high sensitivity (31/32 true positives; 96.8%); the only sample that was missed by the FLOS-LAMP assay had a low number of viral copies, as indicated by its Cq value of 39.3. The high specificity (0/52 false positives; 100%) of the VZV62 FLOS-LAMP assay is attributable to the unique self-quenching/de-quenching property of the target-specific VZV62LPF-FAM probe. The VZV62LPF-FAM probe, which plays a dual role- as an amplifying primer and a reporter probe, can only bind to its target, the dumbbell shaped DNA structure^7^ (Fig. 4). This unique DNA structure is formed only in presence of the target DNA (*ORF62*) through a concerted interplay between the VZV62F3/VZV62B3 and VZV62FIP/VZV62BIP primers (Fig. 4). Any non-dumbbell shaped DNA structure structure(s) which are presumably formed in a typical LAMP reaction, is unable to offer a binding site to the VZV62LPF-FAM probe. As a result, no spurious signal gets generated. This is a major technical advancement considering the fact that the majority of the real-time LAMP assays used in clinical diagnostics use the in-direct detection approach (e.g. intercalating dyes)^14,15^ which cannot discriminate between a genuine amplicon and background. As a result, some form of additional, post-amplification, confirmatory step is usually implemented, for e.g. dissociation curve analysis^32^, to confirm the veracity of the detected signal. Such post-amplification analysis can be a challenge to implement especially in a POC setting, where any unreasonable complications to interpret the data can negatively affect the turnaround time. Moreover, many instruments, including those in the ESEQuant TS2.X family that were used in the present study, lack such confirmatory step functionality. The use of FLOS-LAMP obviates such post-amplification processing, resulting in greater simplicity as well as accuracy.

Based on the result obtained from the VZV62 FLOS-LAMP assay, we converted loop primers of other LAMP primer sets with the following fluorophores- FAM (HSV1-US4, pUC19 and UL54-CMV), and JOE, ROX (pUC19). We observed robust target amplification with these primer sets which suggests that the FLOS probe assay could be adaptable to any LAMP primer set, provided the loop primers have the candidate T residue for fluorophore attachment (see Supplementary Fig. S4). We also believe that FLOS probe technology could be applicable to other iNAAT’s, including HDA^5^, RPA^6^, polymerase spiral reaction (PSR)^33^, cross-priming amplification (CPA)^34^, transcript-based amplification system (TAS)^35^, single primer isothermal amplification (SPIA)^36^, and ramified rolling circle amplification (RAM)^37^. In RPA for example, currently the fluorogenic probe is designed with an apurinic site which, maintains the fluorophore and quencher in close proximity. Upon binding, the apurinic site is recognized by an endonuclease which results in physical separation of the FRET based quenching of the fluorophore. A simpler alternative could be the conversion of either the forward or reverse primer into a self-quenching/dequenching probe by direct internal labelling of the T residue.

One drawback however of our FLOS probe technology is the labelling criterion which must be satisfied if any loop primer needs to be converted into a FLOS-LAMP probe. While no work-around is possible for a loop primer that does not contain the candidate T residue, this can be easily addressed while designing a LAMP primer set *de novo*. In our experience, the PrimerExplorer™ V4 software (Eiken Chemical Co., Ltd., Tokyo, Japan) invariably generates multiple sets of loop primers, allowing a greater flexibility in selecting loop primers which are candidate for FLOS-LAMP. An add-on software utility within the PrimerExplorer™ V4 software, would be an ideal solution to automate this selection process.

In summary, the FLOS-LAMP assay has all the features that are highly sought after in clinical diagnostics and disease management- rapidity, simplicity, sensitivity and specificity. Other features like: (1) pre-assembly and storage of the enzyme-primer/FLOS probe mixture at −80 °C for prolonged periods of time, and (2) tolerance of the FLOS LAMP method to semi-purified templates, makes FLOS-LAMP an ideal iNAAT for clinical diagnostics, where absolute specificity and rapid turnaround time are critical for patient care. The ability to use different fluorophores (FAM, JOE and ROX) further enhances the versatility of this system, and may help in the development of a robust real-time multiplex LAMP for POC setting, an application that has so far proven very challenging to accomplish.

## Methods

### Design of FLOS-LAMP probes

FLOS-LAMP probes were designed by labelling an internal thymine (T) residue of each individual LAMP primer (F3, B3, FIP, BIP, LPB and LPF) with a fluorophore. To identify the exact T residue to which the fluorophore can be attached for efficient quenching/de-quenching, the following labelling criteria^26,27^ were used: (1) presence of cytosine (C) or guanine (G) residue at the terminal 3′ end, (2) T residue at the second or third position from this 3′ end, and (3) one or more G nucleotides flanking the T residue (optional). Initial hypothesis testing and subsequent development/optimization studies focused on a single LAMP primer set, the “VZV62” set (Table 1), which was previously designed to specifically amplify the *ORF62* gene, specific to the alphaherpesevirus Varicella-zoster virus^38^. Based on the results obtained from this study, FLOS-LAMP probes were developed for three other LAMP primer sets for the cytomegalovirus (CMV) gene UL54^39^ (see Supplementary Table S1), herpes simplex virus (HSV)-1 gene US4^40^ (see Supplementary Table S2), and the cloning vector pUC19 (in-house design; see Supplementary Table S3). The VZV62, CMV-UL54 and HSV1-US4 LAMP primer sets were labelled with the commonly used fluorophore, fluorescein (FAM), whereas for the pUC19 LAMP primer set, the pUC19A4LB primer was separately labelled with the fluorophore JOE (6-carboxy-4′, 5′-dichloro-2′,7′-dimethoxyfluorescein). Fluorophores were attached to the T residue by a 6-carbon spacer arm, at position 5 of the cyclic ring. FLOS-LAMP probes were custom ordered (HPLC purification grade) either from IDT (Coralville, IA) or BioBasic Inc. (Markham, ON).

### Target DNA template

The target for VZV62 LAMP set, the ORF62 gene^38^, was ordered in plasmid (Minigene) format from IDT (Coralville, IA). The DNA plasmid (pVZV-ORF62) was dissolved in TE buffer at a working concentration of 1 × 10^6^ copies/µL. The common cloning vector pUC19 was used as a standard template at 1 × 10^6^ copies/µL concentration. A linear double-stranded DNA fragment of the HSV1 US4 gene^40^ was custom synthesised in the gBlock™ (IDT) format and used as the target for the HSV1-US4 LAMP primer set (1 × 10^6^ copies/µL).

### Reaction conditions and Detection of FLOS-LAMP

The FLOS-LAMP reaction (total 20 µL) contained 12 µL of ISO-004nd Isothermal Master Mix (Optigene, Horsham, United Kingdom) containing *Geobacillus* species DNA polymerase (GspSSD LF 2.0), thermostable inorganic pyrophosphatase, optimized buffer (including MgCl_2_, dNTP’s and other proprietary additives), 3 µL of primer/probe mixture and 5 µL of DNA template. In certain experiments, the plus dye version (ISO-004), which contains a proprietary double-stranded DNA intercalating dye was also used. The LAMP assay was run on the real-time fluorimeter, the ESEQuant TS2.6 (Qiagen GmbH, Lake Constance, Germany) instrument at 65 °C for 20 minutes, the optimal time and temperature as recommended by the manufacturer (Optigene). The instrument was programed to acquire the fluorescence signal every 30 sec. Amplification was flagged positive by the ESEQuant TS2 studio software ver 1.17.0 (Qiagen) software when any signal attained slope value, >20 mV from the baseline (0 mV). The time-to-positivity (Tp) value (minutes) was obtained by dividing the numerical value on the x-axis scale, designated as Time[30 sec Interval] by a factor of 2.

### Performance of VZV62 FLOS-LAMP

#### Clinical samples

Clinical samples previously tested positive for VZV either via qPCR^41^ (Cq < 40 = positive; Cq ≥ 40 = negative; see Supplementary Table S4) or a commercial direct fluorescence-assay (DFA; SimFluor; Light Diagnostics™; EMD Millipore) were used to validate the amplification performance of the optimized VZV62 FLOS-LAMP assay. These retrospective, residual samples, stored at −80 **°**C were previously submitted to the Microbiology & Virology Laboratory of the BC Children’s Hospital. These samples were stored in the original universal transport medium (UTM; Copan Diagnostics, Murrieta, CA) in which they were collected. To maintain patient anonymity, each sample was coded and all patient identifiers removed to ensure that personnel involved in the present study were unaware of any patient information. Because the study involved only secondary use of anonymous human biological materials, it was exempted from review by the University of British Columbia Research Ethics Board. DNA was extracted using the InstaGene™ matrix (Bio-Rad Laboratories, Hercules, CA) as follows: equal volume of the sample (50 µL) and InstaGene™ matrix (50 µL) was briefly vortexed and heated at 95 °C for 10 minutes. Five µL of the extract was directly used for LAMP. Non-target micro-organisms consisted of clinical specimens (n = 32), tested positive for HSV-1 and -2, CMV, Epstein-Barr virus (EBV), respiratory syncytial virus (RSV), parainfluenza (Pf) viruses, *Bordetella pertussis* and *Mycoplasma pneumoniae* (Table 5S). The samples were of different types, ranging from nasopharyngeal (NP)/oropharyngeal (OP) swabs, bronchoalveolar lavage (BAL) fluid, urine, saliva, and nasal washes. Two commercial standards, purified human genomic DNA and CMV WHO standard, were also included in the non-target panel (see Supplementary Table S5). Clinical samples previously tested negative for VZV by qPCR were also tested by VZV62 FLOS-LAMP assay (n = 18).

#### Assay Sensitivity Analysis

To determine the limit of detection, a 10-fold serial dilution series of the standard pVZV-ORF62 plasmid (1 × 10^6^ copies/µL) was made in TE buffer and 5 µL was used for VZV62 FLOS-LAMP. Dye-based detection was also performed in parallel by using the intercalating dye version (ISO-004) of the same mastermix, used for VZV62 FLOS-LAMP. The assay was run in triplicate for both VZV62 FLOS and intercalating dye based detection assay.

### Data availability statement

The datasets generated during and/or analyzed during the current study are available from the corresponding author on reasonable request.

## Electronic supplementary material

Supplementary Fig & Tables
