# Supplementary material for: Real-time Detection and Monitoring of Loop Mediated Amplification (LAMP) Reaction Using Self-quenching and De-quenching Fluorogenic Probes
Source: Sci Rep. 2018 Apr 3;8:5548. doi: 10.1038/s41598-018-23930-1 (PMC5883045; doi:10.1038/s41598-018-23930-1)
Supplement: Supplementary file 1 — Supplementary Fig & Tables [file 41598_2018_23930_MOESM1_ESM.pdf]

Real-time Detection and Monitoring of Loop Mediated Amplification (LAMP) Reaction Using Self-quenching and De-quenching Fluorogenic Probes

Vijay J. Gadkar*[1], David M. Goldfarb[1], Soren Gantt[1,2] & Peter A. G. Tilley[1]

[1]Department of Pathology & Laboratory Medicine, Division of Microbiology, Virology & Infection Control
[2]Department of Pediatrics, Division of Infectious Diseases, University of British Columbia and Children's & Women's Health Center of British Columbia, 4500 Oak St, Vancouver V6H 3N1, Canada

Correspondence should be addressed to:
Name: Vijay J. Gadkar, Ph.D.
Email: vijay.gadkar@cw.bc.ca
Tel: (604) 875-2000 (Ext: 7490)
Fax: (604)-875-3777

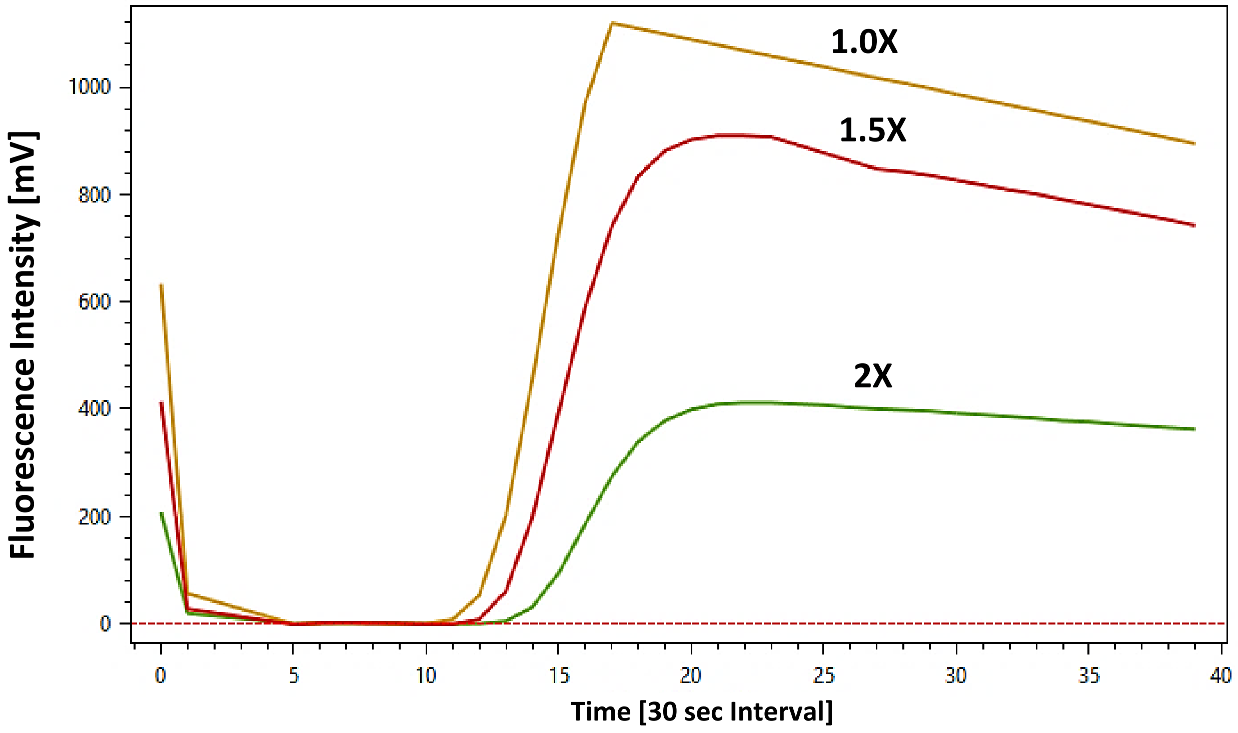

Supplementary Fig. 1S: Change in signal amplitude by increasing levels of Mix7$_{opt}$. Signal amplitude is decreased when the level of Mix7$_{OPT}$ is changed from the optimal 1X to 1.5X and 2X. Each Time[30 sec Interval] (X-axis) represents 30 sec on the ESEQuant instrument.

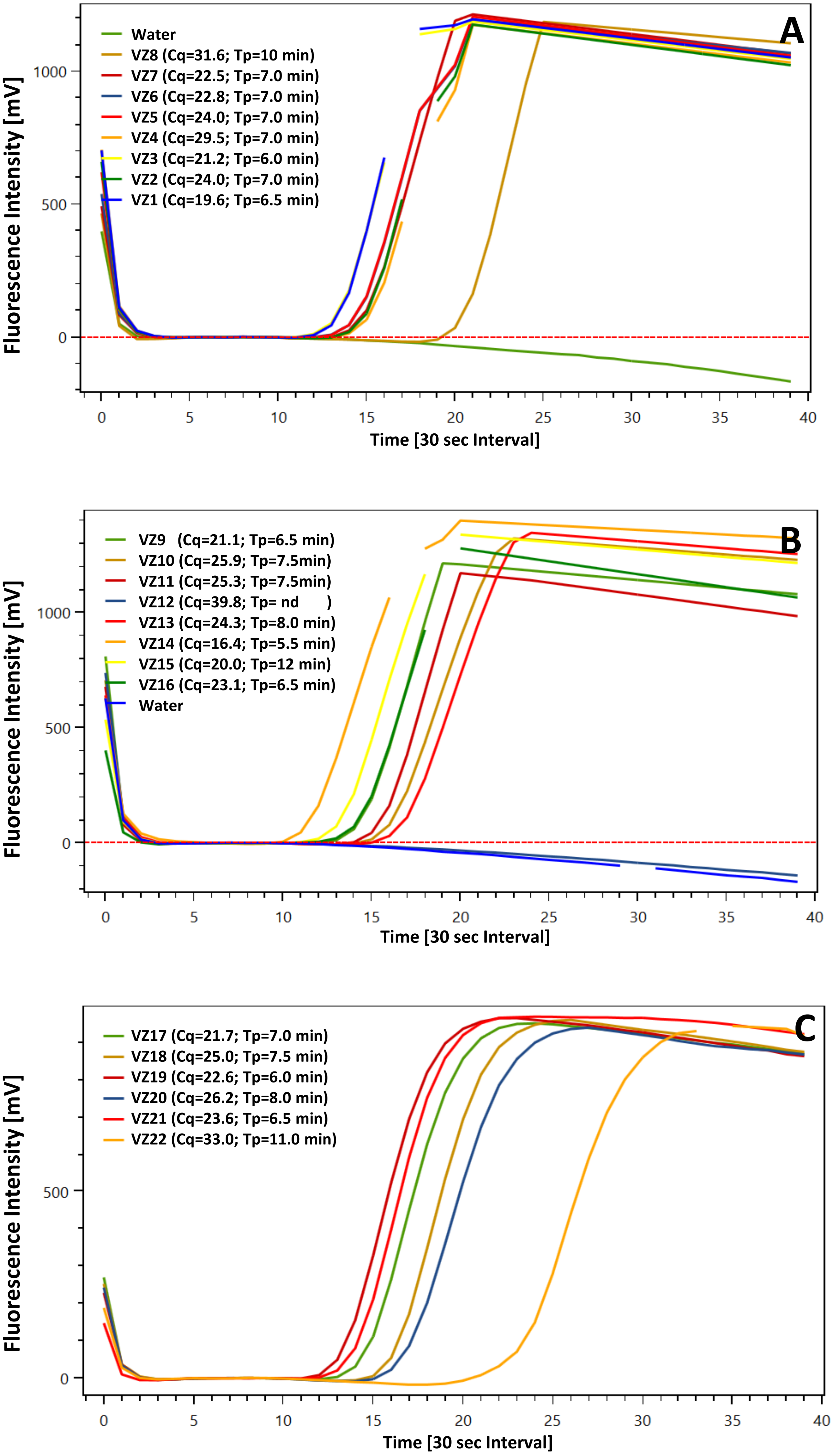

Supplementary Fig. 2S: Detection of VZV from qPCR positive clinical samples using VZV62 FLOS-LAMP assay. Clinical samples positive by VZV qPCR detected by VZV62 FLOS-LAMP assay. Cq and Tp (mint) values against each sample number denotes the original Cq obtained qPCR and FLOS-LAMP respectively. nd= not detected. Each Time[30 sec Interval] (X-axis) represents 30 sec on the ESEQuant instrument.

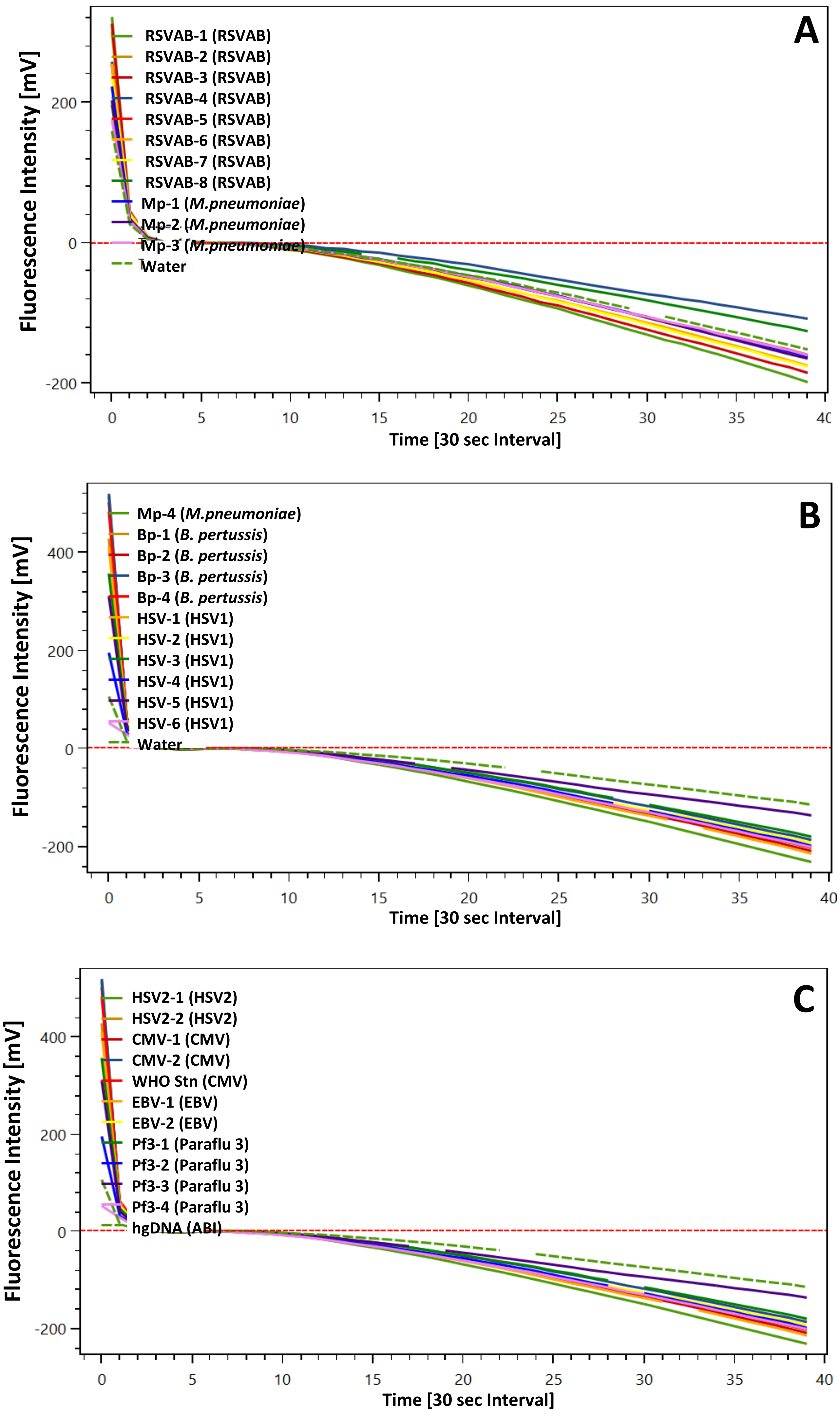

Supplementary Fig. 3S: Specificity analysis of VZV62 FLOS-LAMP assay. Clinical samples positive by different gold standard assays for (**A**.) RSV and *Mycoplasma pneumoniae*, (**B**.) *Mycoplasma pneumoniae, Bordetella pertussis*, and HSV-1, and (**C**.) HSV-2, CMV, EBV, and Pf were all negative by VZV62 FLOS-LAMP. No positive fluorescence signal was detected above the baseline (0 mV) any of these samples. Each Time[30 sec Interval] (X-axis) represents 30 sec on the ESEQuant instrument.

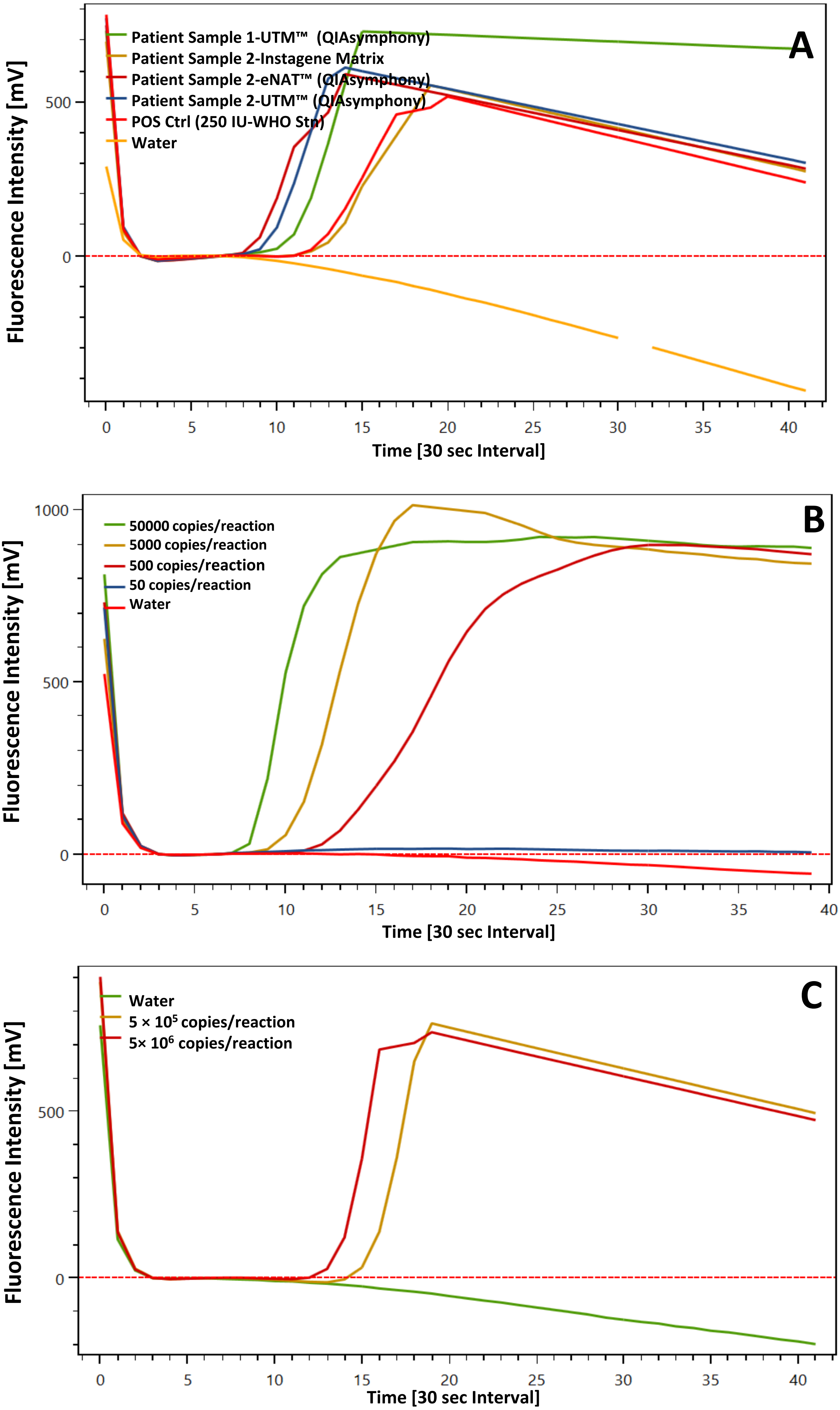

Supplementary Fig. 4S: FLOS-LAMP assay on other LAMP primer sets. (**A.**) Detection of CMV from clinical samples tested positive with qPCR using CMV-UL54 FLOS-LAMP assay. Patient Sample 1, Patient Sample 2, Patient Sample 2 (stored in eNAT™), Patient Sample 2 (stored in UTM™), CMV positive control (250 IU of WHO standard per 20µL reaction volume). UL54LB-FAM probe used at 0.2 µM. (**B.**) pUC19 FLOS-LAMP assay using pUC19A4LB-JOE probe (0.05 µM). pUC19 plasmid template ($5{\times}10^4$, $5{\times}10^3$, $5{\times}10^2$, and 5×10 copies per 20µL reaction volume) and (**C.**) HSV1 FLOS-LAMP assay using HS1LPF-FAM probe (0.2 µM). HSV1 gBlock™ template ($5{\times}10^6$ and $5{\times}10^5$ copies per 20µL reaction volume). Water was used as a negative control in each assay. Each Time[30 sec Interval] (X-axis) represents 30 sec on the ESEQuant instrument.

TARGET	PRIMER NAME	SEQUENCE
CMV UL54 gene	**UL54 F3**	CTCGCCCGAAAACGTGTC
	**UL54 B3**	CGCTGGCAATGACCTCAC
	**UL54 FIP**	CTGTGGCCCCGAAGAAACGCCGCGCTATCGTCAGCATC
	**UL54 BIP**	TCTGCGTCAACGTTTTCGGGCCCTATCGGTGTCGCTGTACT
	**UL54 LF**	ACACGTTCCCCGAAGGC
	**UL54 LB**	AGCGCAGCTACTTTTACTG<u>T</u>G

Supplementary Table. 1S: CMV-UL54 LAMP primer set. <u>T</u> denotes the site for FAM attachment.

TARGET	PRIMER NAME	SEQUENCE
HSV1 ORF US4 (glycoprotein G)	**HS1F3**	GCCGTTGTTCCCATTATCCC
	**HS1B3**	TACTTGGCATGGGGGGTG
	**HS1FIP**	GTTGGGTGGTGGAGGAGACGTCCTTTTGGTTCTTGTCGGT
	**HS1BIP**	GGTCGTCCCTCGCATGAAGCGGCGTGGTAAGGCTGATG
	**HS1LPF**	TTGGTGGGAACCCCCGA<u>**T**</u>AC
	**HS1LPB**	AACATGACCCAGACCGGCAC

Supplementary Table. 2S: HSV1 LAMP primer set. <u>T</u> denotes the site for FAM attachment.

TARGET	PRIMER NAME	SEQUENCE
pUC19 Plasmid	**PUC19A4F3**	GCGGCCAACTTACTTCTGAC
	**PUC19A4B3**	CAACGTTGTTGCCATTGCTA
	**PUC19A4FIP**	AGGCGAGTTACATGATCCCCCAGATCGGAGGACCGAAGGAG
	**PUC19A4BIP**	TGATCGTTGGGAACCGGAGCCAGGCATCGTGGTGTCAC
	**PUC19A4LB**	TGTTGTGCAAAAAAGCGGT**T**AG
	**PUC19A4LF**	TGAAGCCATACCAAACGACG

Supplementary Table. 3S: pUC19 LAMP primer set. T denotes the site for JOE or ROX attachment.

No	Sample Code	Target Organism	Tested Positive via	Extraction Method	VZV62 FLOS-LAMP
1.	RSVAB-1	RSV AB	DFA[1]	SpeedXtract Virus kit (Qiagen)	NEGATIVE
2.	RSVAB-2	RSV AB	DFA[1]	SpeedXtract Virus kit (Qiagen)	NEGATIVE
3.	RSVAB-3	RSV AB	DFA[1]	SpeedXtract Virus kit (Qiagen)	NEGATIVE
4.	RSVAB-4	RSV AB	DFA[1]	SpeedXtract Virus kit (Qiagen)	NEGATIVE
5.	RSVAB-5	RSV AB	DFA[1]	SpeedXtract Virus kit (Qiagen)	NEGATIVE
6.	RSVAB-6	RSV AB	DFA[1]	SpeedXtract Virus kit (Qiagen)	NEGATIVE
7.	RSVAB-7	RSV AB	DFA[1]	SpeedXtract Virus kit (Qiagen)	NEGATIVE
8.	RSVAB-8	RSV AB	DFA[1]	SpeedXtract Virus kit (Qiagen)	NEGATIVE
9.	Mp-1	*Mycoplasma pneumoniae*	qPCR[2]	Instagene Matrix (Bio-Rad)	NEGATIVE
10.	Mp-2	*Mycoplasma pneumoniae*	qPCR[2]	Instagene Matrix (Bio-Rad)	NEGATIVE
11.	Mp-3	*Mycoplasma pneumoniae*	qPCR[2]	Instagene Matrix (Bio-Rad)	NEGATIVE
12.	Mp-4	*Mycoplasma pneumoniae*	qPCR[2]	Instagene Matrix (Bio-Rad)	NEGATIVE
13.	Bp-1	*Bordetella pertusis*	qPCR[3]	Instagene Matrix (Bio-Rad)	NEGATIVE
14.	Bp-2	*Bordetella pertusis*	qPCR[3]	Instagene Matrix (Bio-Rad)	NEGATIVE
15.	Bp-3	*Bordetella pertusis*	qPCR[3]	Instagene Matrix (Bio-Rad)	NEGATIVE
16.	Bp-4	*Bordetella pertusis*	qPCR[3]	Instagene Matrix (Bio-Rad)	NEGATIVE
17.	HSV1-1	HSV1	qPCR[4]	QIAsymphony (Qiagen)	NEGATIVE
18.	HSV1-2	HSV1	qPCR[4]	QIAsymphony (Qiagen)	NEGATIVE
19.	HSV1-3	HSV1	qPCR[4]	QIAsymphony (Qiagen)	NEGATIVE
20.	HSV1-4	HSV1	qPCR[4]	QIAsymphony (Qiagen)	NEGATIVE
21.	HSV1-5	HSV1	qPCR[4]	QIAsymphony (Qiagen)	NEGATIVE
22.	HSV1-6	HSV1	qPCR[4]	QIAsymphony (Qiagen)	NEGATIVE
23.	HSV2-1	HSV2	qPCR[4]	QIAsymphony (Qiagen)	NEGATIVE
24.	HSV2-2	HSV2	qPCR[4]	QIAsymphony (Qiagen)	NEGATIVE
25.	CMV-1	CMV	qPCR[5]	Instagene Matrix (Bio-Rad)	NEGATIVE
26.	CMV-2	CMV	qPCR[5]	Instagene Matrix (Bio-Rad)	NEGATIVE
27.		CMV (WHO standard) 5×10^4 IU/m			NEGATIVE
28.	EBV-1	EBV	qPCR[6]	QIAsymphony (Qiagen)	NEGATIVE
29.	EBV-2	EBV	qPCR[6]	QIAsymphony (Qiagen)	NEGATIVE
30	Pf3-1	Paraflu 3	RT-qPCR[7]	QIAsymphony (Qiagen)	NEGATIVE
31.	Pf3-2	Paraflu 3	RT-qPCR[7]	QIAsymphony (Qiagen)	NEGATIVE
32.	Pf3-3	Paraflu 3	RT-qPCR[7]	QIAsymphony (Qiagen)	NEGATIVE
33.	Pf3-4	Paraflu 3	RT-qPCR[7]	QIAsymphony (Qiagen)	NEGATIVE
34.		Human Control genomic DNA[8]			NEGATIVE

Supplementary Table 5S: List of targets used for specificity testing in the VZV62 FLOS-LAMP.

1. LIGHT DIAGNOSTICS™ SimulFluor® Respiratory Screen kit (EMD Millipore, USA)
2. Touati et al (2009) J Clin Microbiol. 47(7):2269-71.
3. Hasan et al (2014) Can J Infec Dis Med Micro 25(4). 217-221.
4. Corey et al (2005) J Med Virol. 76(3):350-355.
5. In-house TaqMan™ assay
6. In-house TaqMan™ assay
7. Lambert et al (2008) Pediatrics 122(3). e615-620.
8. Supplied with ABI RNase P kit (ABI Cat No: 4316831); Purified, 10 ng/uL
